# Understanding and Evaluating Survey Research

**Published:** 2015-03-01

**Authors:** Julie Ponto

**Affiliations:** Winona State University, Rochester, Minnesota

A variety of methodologic approaches exist for individuals interested in conducting research. Selection of a research approach depends on a number of factors, including the purpose of the research, the type of research questions to be answered, and the availability of resources. The purpose of this article is to describe survey research as one approach to the conduct of research so that the reader can critically evaluate the appropriateness of the conclusions from studies employing survey research.

## SURVEY RESEARCH

Survey research is defined as "the collection of information from a sample of individuals through their responses to questions" (Check & Schutt, 2012, p. 160). This type of research allows for a variety of methods to recruit participants, collect data, and utilize various methods of instrumentation. Survey research can use quantitative research strategies (e.g., using questionnaires with numerically rated items), qualitative research strategies (e.g., using open-ended questions), or both strategies (i.e., mixed methods). As it is often used to describe and explore human behavior, surveys are therefore frequently used in social and psychological research (Singleton & Straits, 2009).

Information has been obtained from individuals and groups through the use of survey research for decades. It can range from asking a few targeted questions of individuals on a street corner to obtain information related to behaviors and preferences, to a more rigorous study using multiple valid and reliable instruments. Common examples of less rigorous surveys include marketing or political surveys of consumer patterns and public opinion polls.

Survey research has historically included large population-based data collection. The primary purpose of this type of survey research was to obtain information describing characteristics of a large sample of individuals of interest relatively quickly. Large census surveys obtaining information reflecting demographic and personal characteristics and consumer feedback surveys are prime examples. These surveys were often provided through the mail and were intended to describe demographic characteristics of individuals or obtain opinions on which to base programs or products for a population or group.

More recently, survey research has developed into a rigorous approach to research, with scientifically tested strategies detailing who to include (representative sample), what and how to distribute (survey method), and when to initiate the survey and follow up with nonresponders (reducing nonresponse error), in order to ensure a high-quality research process and outcome. Currently, the term "survey" can reflect a range of research aims, sampling and recruitment strategies, data collection instruments, and methods of survey administration.

Given this range of options in the conduct of survey research, it is imperative for the consumer/reader of survey research to understand the potential for bias in survey research as well as the tested techniques for reducing bias, in order to draw appropriate conclusions about the information reported in this manner. Common types of error in research, along with the sources of error and strategies for reducing error as described throughout this article, are summarized in the Table.

**Table 1:**
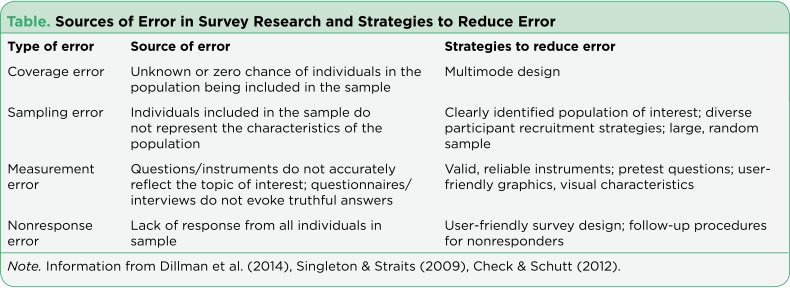
Sources of Error in Survey Research and Strategies to Reduce Error

## SAMPLING

The goal of sampling strategies in survey research is to obtain a sufficient sample that is representative of the population of interest. It is often not feasible to collect data from an entire population of interest (e.g., all individuals with lung cancer); therefore, a subset of the population or sample is used to estimate the population responses (e.g., individuals with lung cancer currently receiving treatment). A large random sample increases the likelihood that the responses from the sample will accurately reflect the entire population. In order to accurately draw conclusions about the population, the sample must include individuals with characteristics similar to the population.

It is therefore necessary to correctly identify the population of interest (e.g., individuals with lung cancer currently receiving treatment vs. all individuals with lung cancer). The sample will ideally include individuals who reflect the intended population in terms of all characteristics of the population (e.g., sex, socioeconomic characteristics, symptom experience) and contain a similar distribution of individuals with those characteristics. As discussed by Mady Stovall beginning on page 162, Fujimori et al. (2014), for example, were interested in the population of oncologists. The authors obtained a sample of oncologists from two hospitals in Japan. These participants may or may not have similar characteristics to all oncologists in Japan.

Participant recruitment strategies can affect the adequacy and representativeness of the sample obtained. Using diverse recruitment strategies can help improve the size of the sample and help ensure adequate coverage of the intended population. For example, if a survey researcher intends to obtain a sample of individuals with breast cancer representative of all individuals with breast cancer in the United States, the researcher would want to use recruitment strategies that would recruit both women and men, individuals from rural and urban settings, individuals receiving and not receiving active treatment, and so on. Because of the difficulty in obtaining samples representative of a large population, researchers may focus the population of interest to a subset of individuals (e.g., women with stage III or IV breast cancer). Large census surveys require extremely large samples to adequately represent the characteristics of the population because they are intended to represent the entire population.

## DATA COLLECTION METHODS

Survey research may use a variety of data collection methods with the most common being questionnaires and interviews. Questionnaires may be self-administered or administered by a professional, may be administered individually or in a group, and typically include a series of items reflecting the research aims. Questionnaires may include demographic questions in addition to valid and reliable research instruments (Costanzo, Stawski, Ryff, Coe, & Almeida, 2012; DuBenske et al., 2014; Ponto, Ellington, Mellon, & Beck, 2010). It is helpful to the reader when authors describe the contents of the survey questionnaire so that the reader can interpret and evaluate the potential for errors of validity (e.g., items or instruments that do not measure what they are intended to measure) and reliability (e.g., items or instruments that do not measure a construct consistently). Helpful examples of articles that describe the survey instruments exist in the literature (Buerhaus et al., 2012).

Questionnaires may be in paper form and mailed to participants, delivered in an electronic format via email or an Internet-based program such as SurveyMonkey, or a combination of both, giving the participant the option to choose which method is preferred (Ponto et al., 2010). Using a combination of methods of survey administration can help to ensure better sample coverage (i.e., all individuals in the population having a chance of inclusion in the sample) therefore reducing coverage error (Dillman, Smyth, & Christian, 2014; Singleton & Straits, 2009). For example, if a researcher were to only use an Internet-delivered questionnaire, individuals without access to a computer would be excluded from participation. Self-administered mailed, group, or Internet-based questionnaires are relatively low cost and practical for a large sample (Check & Schutt, 2012).

Dillman et al. (2014) have described and tested a tailored design method for survey research. Improving the visual appeal and graphics of surveys by using a font size appropriate for the respondents, ordering items logically without creating unintended response bias, and arranging items clearly on each page can increase the response rate to electronic questionnaires. Attending to these and other issues in electronic questionnaires can help reduce measurement error (i.e., lack of validity or reliability) and help ensure a better response rate.

Conducting interviews is another approach to data collection used in survey research. Interviews may be conducted by phone, computer, or in person and have the benefit of visually identifying the nonverbal response(s) of the interviewee and subsequently being able to clarify the intended question. An interviewer can use probing comments to obtain more information about a question or topic and can request clarification of an unclear response (Singleton & Straits, 2009). Interviews can be costly and time intensive, and therefore are relatively impractical for large samples.

Some authors advocate for using mixed methods for survey research when no one method is adequate to address the planned research aims, to reduce the potential for measurement and non-response error, and to better tailor the study methods to the intended sample (Dillman et al., 2014; Singleton & Straits, 2009). For example, a mixed methods survey research approach may begin with distributing a questionnaire and following up with telephone interviews to clarify unclear survey responses (Singleton & Straits, 2009). Mixed methods might also be used when visual or auditory deficits preclude an individual from completing a questionnaire or participating in an interview.

## FUJIMORI ET AL.: SURVEY RESEARCH

Fujimori et al. (2014) described the use of survey research in a study of the effect of communication skills training for oncologists on oncologist and patient outcomes (e.g., oncologist’s performance and confidence and patient’s distress, satisfaction, and trust). A sample of 30 oncologists from two hospitals was obtained and though the authors provided a power analysis concluding an adequate number of oncologist participants to detect differences between baseline and follow-up scores, the conclusions of the study may not be generalizable to a broader population of oncologists. Oncologists were randomized to either an intervention group (i.e., communication skills training) or a control group (i.e., no training).

Fujimori et al. (2014) chose a quantitative approach to collect data from oncologist and patient participants regarding the study outcome variables. Self-report numeric ratings were used to measure oncologist confidence and patient distress, satisfaction, and trust. Oncologist confidence was measured using two instruments each using 10-point Likert rating scales. The Hospital Anxiety and Depression Scale (HADS) was used to measure patient distress and has demonstrated validity and reliability in a number of populations including individuals with cancer (Bjelland, Dahl, Haug, & Neckelmann, 2002). Patient satisfaction and trust were measured using 0 to 10 numeric rating scales. Numeric observer ratings were used to measure oncologist performance of communication skills based on a videotaped interaction with a standardized patient. Participants completed the same questionnaires at baseline and follow-up.

The authors clearly describe what data were collected from all participants. Providing additional information about the manner in which questionnaires were distributed (i.e., electronic, mail), the setting in which data were collected (e.g., home, clinic), and the design of the survey instruments (e.g., visual appeal, format, content, arrangement of items) would assist the reader in drawing conclusions about the potential for measurement and nonresponse error. The authors describe conducting a follow-up phone call or mail inquiry for nonresponders, using the Dillman et al. (2014) tailored design for survey research follow-up may have reduced nonresponse error.

## CONCLUSIONS

Survey research is a useful and legitimate approach to research that has clear benefits in helping to describe and explore variables and constructs of interest. Survey research, like all research, has the potential for a variety of sources of error, but several strategies exist to reduce the potential for error. Advanced practitioners aware of the potential sources of error and strategies to improve survey research can better determine how and whether the conclusions from a survey research study apply to practice.
